# Biomechanical spinal growth modulation and progressive adolescent scoliosis – a test of the 'vicious cycle' pathogenetic hypothesis: Summary of an electronic focus group debate of the IBSE

**DOI:** 10.1186/1748-7161-1-16

**Published:** 2006-10-18

**Authors:** Ian AF Stokes, R Geoffrey Burwell, Peter H Dangerfield

**Affiliations:** 1 Department of Orthopaedics and Rehabilitation, University of Vermont, Burlington, Vermont 05405, USA; 2 The Centre for Spinal Studies & Surgery, Queen's Medical Centre, Nottingham, NG7 2UH, UK; 3 Sherrington Buildings, Ashton Street, Liverpool, L69 3GE, UK

## Abstract

There is no generally accepted scientific theory for the causes of adolescent idiopathic scoliosis (AIS). As part of its mission to widen understanding of scoliosis etiology, the International Federated Body on Scoliosis Etiology (IBSE) introduced the electronic focus group (EFG) as a means of increasing debate on knowledge of important topics. This has been designated as an on-line Delphi discussion. The text for this debate was written by Dr Ian A Stokes. It evaluates the hypothesis that in progressive scoliosis vertebral body wedging during adolescent growth results from asymmetric muscular loading in a "vicious cycle" (*vicious cycle hypothesis of pathogenesis*) by affecting vertebral body growth plates (endplate physes). A frontal plane mathematical simulation tested whether the calculated loading asymmetry created by muscles in a scoliotic spine could explain the observed rate of scoliosis increase by measuring the vertebral growth modulation by altered compression. The model deals only with vertebral (not disc) wedging. It assumes that a pre-existing scoliosis curve initiates the mechanically-modulated alteration of vertebral body growth that in turn causes worsening of the scoliosis, while everything else is anatomically and physiologically 'normal' The results provide quantitative data consistent with the *vicious cycle hypothesis*. Dr Stokes' biomechanical research engenders controversy. A new speculative concept is proposed of vertebral symphyseal dysplasia with implications for Dr Stokes' research and the etiology of AIS. What is not controversial is the need to test this hypothesis using additional factors in his current model and in three-dimensional quantitative models that incorporate intervertebral discs and simulate thoracic as well as lumbar scoliosis. The growth modulation process in the vertebral body can be viewed as one type of the biologic phenomenon of *mechanotransduction*. In certain connective tissues this involves the effects of mechanical strain on chondrocytic metabolism a possible target for novel therapeutic intervention.

## Background

In the absence of any generally accepted scientific theory for the etiology of idiopathic scoliosis, treatment remains pragmatic with a very incomplete scientific basis. The International Federated Body on Scoliosis Etiology (IBSE) [1] introduced the Electronic Focus Group (EFG) as a means of increasing debate of knowledge on important topics. The text for this debate was written by Dr Ian Stokes who addresses the concept of mechanical modulation of vertebral body growth in the pathogenesis of progressive adolescent scoliosis generally attributed to the Hueter-Volkmann or Delpech effect [1-25] in which constant pathologic strong pressure inhibits endochondral longitudinal growth while reduced compression accelerates growth [2-4]; pressure exerted eccentrically causes an active change in the direction of growth [2,5-7]. Brace treatment is based on this effect although the efficacy of bracing continues to be debated and questioned [26-39] while exercises are not even considered by many clinicians. Guidelines for the conservative management of scoliosis by physical therapy, intensive rehabilitation and brace treatment have been published recently [37,38]; on *evidence-based medicine *[40,41] bracing and exercises gave a grade of evidence C (level of evidence IV)[37] (i.e. expert committee reports or opinions and/or clinical experience of respected authorities indicating that directly applicable clinical studies of good quality are absent). Since it is generally recognized that multi-level arthrodesis of the spine is not a desirable outcome, currently there is renewed interest in modifying vertebral growth by early surgical interventions [42-44] including stapling and, in young children, fusionless scoliosis surgery [45] which is being evaluated experimentally in animals [46-48].

The mechanical modulation of vertebral growth in the presumed asymmetrically loaded spine with scoliosis was described by Stokes as a *'vicious cycle' *and interpreted by his *vicious cycle hypothesis of pathogenesis *[3,4]. Roaf [10] had previously used the term *'vicious circle' *to describe the effects of gravity on thoracic vertebral endplate physes in Scheuermann's disease whatever the primary cause of that deformity. The implication is that independent of whether a scoliosis is congenital, neuromuscular, or idiopathic, mechanical factors become predominant relative to initiating factors during rapid adolescent growth, when the risk of curve progression is greatest [49-51]. While qualitatively attractive, the validity of this *mechanical stress-growth relationship hypothesis *remains to be proven. Proof requires quantitative information about the loading state of the spine with scoliosis, consequential growth alterations and geometrical changes all of which are addressed here.

The Statement of this EFG is drawn from biomechanical studies that Dr Stokes has pursued in recent years relating to a mathematical simulation of the *vicious cycle hypothesis *for the pathogenesis of adolescent scoliosis. A frontal plane model was designed because very little is known about the details of how the loads are transmitted through the spine in three-dimensions. The model, involving geometric recursive/growth analysis, tested whether (1) the calculated loading asymmetry of a growing spine with scoliosis created by a *neuromuscular activation strategy*, together with (2) the measured bone growth sensitivity to altered compression could explain (3) the observed rate of scoliosis progression during adolescent growth. The model assumes that (1) a pre-existing scoliosis curve of unknown etiology initiates the mechanically modulated alteration of growth that in turn causes worsening of the scoliosis, (2) everything else is anatomically and physiologically 'normal', and (3) loading sustained over a substantial proportion of the day modulates endochondral bone growth, whereas transient loading does not. The forces due to muscle activation are generally of greater magnitude than forces due to superimposed body weight [52]. The simulations employ newly-available data on the magnitude of asymmetric loading imposed on the spine as a function of the scoliosis curve, and of the resulting mechanically altered vertebral growth. The effects of intervertebral disc wedging were included only indirectly by prior knowledge of their relationship to vertebral wedging. The findings are consistent with the *vicious cycle hypothesis of pathogenesis *namely that in progressive adolescent idiopathic scoliosis (AIS) frontal plane vertebral body wedging during growth results from asymmetric neuromuscular loading.

The research engenders controversy, including:

1. Are the results applicable to humans who lack ossified vertebral body epiphyses and have "ring" apophyses?

2. Are there separate initiating (? discal, vertebral, costal or neuromuscular) and progressive (mechanical and non-mechanical) factors for AIS pathogenesis?

3. Are vertebral endplate physes normal when the growth modulation starts?

4. Whether healthy adolescents can spontaneously generate asymmetrical vertebral growth and deformity by inappropriate neuromuscular activation strategies.

5. Do the findings have relevance to treatment? Or, is the resurrection of exercise programs for AIS a step too far?

6. Why does asymmetric loading on the spine from pelvic tilt scoliosis not lead to curve progression?

7. Does movement asymmetry of both hips during gait cause idiopathic scoliosis?

8. Why do *normal *sagittal spinal curves not progress from front-back asymmetric vertebral loading?

9. Might not patients with severe curves have, in addition to the hypokyphosis, a slightly postero-lateral asymmetric load on endplate physes?

10. Do neurogenic thoracic scolioses result from different skeletal pathomechanisms than those that evoke thoracic AIS?

11. In some conditions why does curve progression occur without evidence to suggest that the cause is asymmetric loading?

12. Do the relative anterior spinal overgrowth (RASO) and other biologic concepts of structural scoliosis contribute to curve progression?

13. Does the vertebral body wedging in progressive lumbar AIS result from:

a) secondary neuromuscular dysfunction [this paper],

b) primary neuromuscular imbalance [12,19,22,53,54],

c) relative anterior spinal overgrowth (RASO) due to -

i. primary skeletal change [12,55,56] with uncoupled endochondral-membranous bone formation [55,56], or

ii. uncoupled neuro-osseous growth between the anterior spinal column and spinal cord [57-60],

d) calcification of cartilage endplates [61,62],

e) resorption by osteoclasts [63], or

f) osteopenia [64-67], possibly due to maturational abnormalities in cell differentiation – recently suggested by studying calcium channel isoforms in the membranes of platelets and osteoblasts from patients with AIS [68].

14. Do methods and findings from recent research on mechanotransduction in articular cartilage have relevance to the vertebral growth plate chondrocytic phenotype?

15. Is the adjective "vicious" appropriate for Dr Stokes' biomechanical hypothesis of pathogenesis?

A new speculative concept is proposed namely of vertebral *symphyseal dysplasia *with implications for Dr Stokes' research and the etiology of AIS. It explains the developmental onset of AIS in morphological, biomechanical and molecular terms and is complementary to a vascular concept of pathogenesis [69-71]. What is not controversial is the need to test the Dr Stokes' hypothesis using additional factors not only in the current model but also in three-dimensional quantitative models that incorporate intervertebral discs and simulate thoracic as well as lumbar scoliosis. An urgent challenge is to be able to distinguish the factors that predict whether a curve is progressive or not.

The vertebral body growth modulation process is one type of the biologic phenomenon of *mechanotransduction *[72-75]. In this phenomenon a single physical parameter – force – is converted into a response that is the basis for a plethora of fundamental biologic processes known to occur in many tissues including skeletal tissues [76,77], muscles, tendons and ligaments [78,79]. There is recent evidence that cells from distinct regions of intervertebral discs of cattle tails differ in their mechanosensitivity as revealed by gene expression [80]. The finding that extracellular matrix genes are upregulated by cyclical mechanical strain suggests that such genes are possible targets for novel therapeutic intervention [81].

In sum, this EFG aims to explore what may be learnt about the etiopathogenesis of AIS by IBSE members debating via e-mail Dr Stokes' biomechanical spinal growth modulation experiments on progressive adolescent scoliosis. Biomechanical, biological and clinical issues are discussed, a novel hypothesis formulated and recent relevant research on AIS etiopathogenesis is considered.

## Statement by Dr Stokes

### Quantitative simulation of vicious cycle hypothesis for scoliosis curve progression

**Figure 1:**
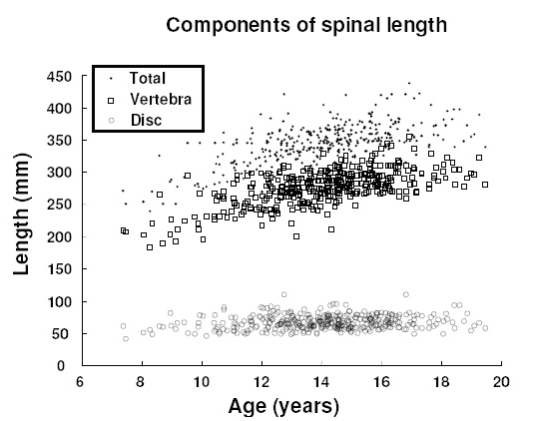
Spinal growth (T3-L5) during adolescence, as shown by a cross-sectional study of spinal length, and the contributions from total vertebral height, and from total disc height. Lengths were obtained from stereoradiographic reconstructions of patients in a scoliosis clinic [83].

In the vicious cycle hypothesis it is proposed that lateral spinal curvature produces asymmetrical loading of the skeletally immature spine, which in turn causes asymmetrical growth and hence progressive wedging deformity. Both discal and vertebral wedging contribute to a scoliosis curvature. The relative contributions of these two structures are not well-defined. Both apparently contribute approximately equally to the lateral spinal curvature [82], but their mechanism of growth differs. In a retrospective cross-sectional examination of three-dimensional reconstructions from stereoradiographs of 208 adolescents with idiopathic scoliosis [83], the total spinal length, and the relative contributions of vertebrae and disc to spinal length were recorded (Figure 1). The spinal growth was apparently due almost entirely to vertebral growth in this adolescent phase of growth, with minimal if any increase in the disc heights.

Human vertebrae grow longitudinally by ossification of vertebral body growth cartilages (endplate physes) similar to long bones but lacking ossified epiphyses other than the "ring" apophyses. The endplate physes adjacent to the discs generate longitudinal growth, while the vertebrae increase in diameter by appositional growth [84,85]. Axial compression reduces the axial growth, apparently through a combination of reduced numbers of proliferating chondrocytes and reduced chondrocytic enlargement in the hypertrophic zone [86]. Biomechanical influences on the postnatal modeling and remodeling of intervertebral discs have not been described, but the underlying mechanisms are probably quite different from those in vertebrae. Wedging of discs in scoliosis may involve asymmetric tissue remodeling or selective concave side degeneration [87].

This Statement presents an analytical simulation of the evolution of a scoliosis curvature, based on the vicious cycle hypothesis, and employing published quantitative estimates of the key variables in this proposed mechanism of curve progression.

## Methods

### Loading of the lumbar spine with scoliosis

The magnitude of level-specific spinal loading asymmetry was estimated for a three-dimensional spinal geometry with several different magnitudes of scoliosis, assuming physiologically plausible *neuromuscular activation strategies *[88]. The strategy that minimized the sum of cubed muscle stresses (a physiologically efficient strategy in terms of energy utilization) was compared with strategies that equalized or reversed the loading asymmetry at the curve apex. The presumed strategies were required to solve the 'redundancy' problem (more muscles than spinal degrees of freedom) in these biomechanical analyses. Muscle activation and spinal loading were calculated for different static tasks, represented by different magnitudes and directions of an external force or moment generated by the modeled person. These external forces were six force directions at 50% and 75% of the corresponding maximum effort, and five moment directions (omitting right axial rotation) [88].

### Load modulated growth of vertebrae

Vertebral and tibial growth plate response to sustained compression was measured in three different animal species with -0.1 MPa (distraction), 0 MPa (sham), 0.1 or 0.2 MPa (compression) of sustained compressive stress applied across each growth plate. The stresses resulted from forces applied by an external apparatus attached to pins passed through the diaphysis and epiphysis. [86,89].

### Spinal growth and simulation of the vicious cycle

The spinal growth during each of the adolescent years from 10 to 16 was estimated from a growth curve generated from spinal length measurements obtained from stereoradiographic studies of 208 adolescent patients with scoliosis [83,90]. The modeled geometry was averaged from stereoradiographic studies of 15 patients with a thoracolumbar Cobb angle in the range 27–43 degrees In the simulations of growth, the initial spinal geometry in two dimensions was defined by a lumbar scoliosis of 26 degrees Cobb angle, averaged and scaled from the 15 patients' radiographs [88]. The estimates of level-specific spinal loading asymmetry, together with the relationship expressing growth sensitivity to load were included in an analysis that was used to estimate the stress distribution across each vertebral endplate, and the resulting asymmetric vertebral growth. The contribution of the altered growth to the progression of a scoliosis curvature was then calculated. This spinal growth simulation was conducted for each of the positive and negative principal force and moment directions for which spinal loading estimates had been performed [88], and for 50% and 75% of maximum effort in turn. The vertical loading direction corresponded to the modeled spine resisting gravity forces. Two additional factors were taken into account: First, since the disc wedging contributes about half of the lateral curvature, the curvature increase was considered to be double the increases due to vertebral wedging. However, assuming that an individual only loads the spine at the simulated levels of effort for half of the 24-hour period, the predicted progression from full-time loading would be halved, since it has been found that diurnal loading (12 hours of loading/day) produced approximately half of the growth modulation effect, relative to full-time loading [86].

## Results

### Spinal loading asymmetry

Spinal loading asymmetry was dependent on the *neuromuscular activation strategy*. In the strategy that is considered most physiological [91], the sum of the cubed muscle stresses was minimized. In this case the spine was loaded more on its concave side at the curve apex in most activities (external loading directions) [88]. However, if the analyses used minimum spinal loading asymmetry as the 'cost function', then symmetrical spinal loading could be achieved, but with a substantial increase in the physiological energy cost. This finding suggests that different individuals may adopt differing neuromuscular activation strategies, with consequences for the spinal loading that could explain why some individuals have more progressive scoliosis curves than others.

### Stress-modulated growth in vertebrae and tibiae

Growth rates at axially loaded growth plates (tail vertebrae and proximal tibiae) were found to be modulated relatively uniformly (independent of anatomical location) and proportional to stress magnitude. The growth data were therefore expressed in a linear formulation of growth G as a function of compressive stress σ, thus:

G = G_m_(1-β(σ-σ_m_))

where β = 1.87 MPa^-1 ^was the empirically determined constant from regression analysis of the experimental data. (The subscript *m *signifies the 'baseline' growth and physiological stress for a spine without scoliosis).

### Scoliosis curve progression – vicious cycle simulation

**Figure 2:**
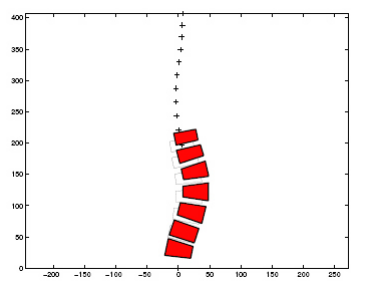
Simulated evolution of the thoracolumbar scoliosis as a result of mechanically modulated asymmetrical growth. The initial geometry (unfilled shapes, and '+' indicating thoracic vertebrae) is the starting geometry at age 11 years (26 degrees Cobb lumbar scoliosis). The final geometry (filled shapes) is averaged from the model-predicted final shapes for all 11 analysed loading directions at age 16 years. Note that only the vertebrae grow asymmetrically and develop progressive wedging in this simulation – the discs do not change shape.

Progression of the scoliosis was predicted by the analytical model in 7 of 11 loading cases at 50% of maximum effort and 10 of 11 loading cases for 75% of maximum efforts. The spinal shape changes were averaged over the 11 loading cases, assuming that daily activity consists of an equal amount of time spent in each activity (Figure 2). Then, for the spine with an initial 26 degrees Cobb scoliosis, the predicted final lumbar spinal curve magnitude was 34 degrees Cobb at age 16 years when the efforts producing the spinal loading were at 50% of maximum effort, and it was 42 degrees Cobb angle when the efforts were at 75% of maximum.

### Illustrative loading scenario

For a single anatomical level at the scoliosis curve apex, the compression force is 1010 N, laterally offset 2 mm from the vertebral center. The associated stresses are calculated as 1.3 MPa (concave side) and 0.7 MPa (convex side). With β = 1.87 MPa^-1^, annual spinal growth of 3% would produce growth of a 30 mm high vertebra of 0.5 mm on the concave side and 1.3 mm on the convex side, implying an increment of angular deformity of 1.15 degrees for a vertebra having a width of 40 mm. Since the apical vertebra contributes about 17% of the Cobb angle, this implies an annual increase in Cobb angle of 6.7 degrees.

## Conclusion

The simulations indicate that a substantial component of scoliosis progression during adolescent growth is biomechanically mediated. It is possible that suitable muscle rehabilitation programs could alter the prevailing spinal loading, since the muscle force analyses [88] indicate that different neuromuscular activation strategies are possible, with differing likelihood of loading the spine asymmetrically. To avoid the unnecessary treatment of non-progressive curves, a means of identifying progressive curves at an early stage is needed.

## General statements

### Comment no. 1

I have long been a proponent of asymmetrical mechanical loads being a factor in the development of abnormal spinal deformity and am pleased to see the theory addressed in such a cogent manner.

### Comment no. 2

Dr. Stokes has developed a strong argument for mechanical asymmetry as a self-directed force of deviation. He makes a point of neuromuscular strategies affecting asymmetric output, but the potential for outcome independent of the strategies used should be considered [Moderator see Comment no. 42].

### Comment no. 3

These data comprise a potential breakthrough that can place scoliosis diagnosis and treatment, long grounded in empirical observation and guesswork, on a solid scientific basis.

### Comment no. 4

This study provides a new quantitative tool for creating progressive and non-progressive models of early curves in growing individuals and for designing new treatments.

### Comment no. 5

I thank Dr. Stokes for this EFG and in previous publications providing much insight into biomechanical spinal growth modulation and how vertebral wedging may develop through the *vicious cycle *mechanism. Dr Stokes' description of the mechanism of biomechanical modulation of scoliosis progression during adolescent growth is fully consistent with our observations on 3D postural unbalancing in scoliotic patients often associated with lower limb length discrepancy and CNS motor control deficiency. It is very stimulating work that helps me understand the phenomena we are recording [92].

### Comment no. 6

The study though excellent in itself must await the clarification of etiology and natural history. I wish I could be more positive, but we continue to run around the same circle (a vicious cycle indeed) of "straight spine acquires pathological posture resulting in secondary remodeling", without any solution, and I am weary of it. Can I be the only one who thinks it is high time we reassessed our basic assumptions? [Moderator see Introduction, controversy item 13 and Comment no.42].

#### Response

Thanks – these are encouraging comments. The *vicious cycle *concept existed previously [Moderator see Introduction] and, as noted by these commentaries, is intuitively and qualitatively a plausible explanation of why scoliosis curves progress. Equally, in the presence of chronic muscle imbalance it might explain the etiology of small curves. However, it has not been quantitatively proven. The challenge I take on is to quantify each step in the *'cycle'*, as it relates to scoliosis progression during growth. This alone does not prove the *vicious cycle hypothesis of pathogenesis*, but adds to its credibility. The implicit question is 'How much of the spinal shape change is due to altered mechanics and growth, and how much is due to other (pre-existing or etiologic) causes?' It should be noted that this paper (1) addresses only the frontal plane, and (2) deals only with vertebral (not discal) wedging.

## Applicability of the findings to humans?

### Comment no. 7

The fact that it is possible to simulate spinal deformity in small animals by various techniques does not prove that this is what occurs in children, or even that growing children are sufficiently analogous to rats or beagle hounds for the comparison to be entertained. It may be so, but it is a big assumption. The simulations are impressive and I do not fault them at all, but they don't prove anything. They calculate the loading on an asymmetric spine, assume that this causes the progression, and find that it is so. It is an elegant but circular argument.

#### Response

Correct. The two crucial components of the argument are: (1) estimates of the loading asymmetry on the spine; and (2) transference of data from controlled animal studies to the human. The former has been estimated analytically (within probable bounds), the latter has been found experimentally in vertebral and tibial growth plates of several species with variable full-time and diurnal loading. As already stated, these analyses add credibility to the *vicious cycle hypothesis *but do not prove it. [Moderator see Refs. [93,94]].

### Comment no. 8

Dr Stokes writes: "The simulations indicate that a substantial component of scoliosis progression during adolescent growth is biomechanically mediated." The artificially-created *near-symmetric loads *in immature tail vertebrae and tibiae of animals were static with sustained compression loading suppressing linear growth more than intermittent loading. These animal growth plates have epiphyses unlike human vertebral bodies that lack epiphyses and have "ring" apophyses [4,12,85,95,96]. Can such data on induced linear growth impairment in animals be applied with confidence to the varying repetitive *eccentric loads *on immature deforming human lumbar vertebrae created during diurnal activities?

#### Response

(1) It is true that our data come from controlled stresses applied uniformly across the growth plates of animals. I believe that the data are applicable to non-uniform stresses applied with diurnal variations to humans, based on our prior studies with asymmetric loads on vertebrae [97] and studies of diurnally varying forces on growth plates [86]. The finding of a very similar proportional growth response to stress across species and anatomical locations [89] adds credibility to the extrapolation of those findings to human vertebrae.

(2) Humans do not have ossified epiphyses between the intervertebral disc and the vertebral body growth plate, and this implies certain differences, notably that the growth plate and disc compete for nutrition from vertebral body blood supply. Mechanically, the stresses on the growth plate are probably not much altered by the presence or absence of a *thin, ossified epiphysis*. I expect that the absence of vertebral epiphyses in humans has greater implications for *disc *metabolism in skeletally immature humans than for their *growth plates *[Moderator see Ref. [93]].

## The hypothesis and secondary effects

### Comment no. 9

This study depends on the hypothesis that *initiating factors *can be meaningfully distinguished from *progressing factors*, and that the latter are mechanical [Moderator see Refs. [19,22]]. There is no evidence for this, although it may be correct. The simulation tested whether "calculated loading asymmetry created by muscles in a spine with scoliosis could explain the observed rate of scoliosis". Enneking and Harrington [98] clearly hoped to find evidence for loading asymmetry (they called it an "attractive speculation") but they did not find it in the inferior articular processes of apical vertebrae excised at surgery.

#### Response

Yes, this study examines the effects on curve progression of asymmetric forces acting on endplate physes that are a consequence of a pre-existing curve, independent of the curve-initiating factors. If asymmetric spinal loading were also present, *e.g*. in neuromuscular scoliosis, then it would also contribute to progression of the curve. The trabeculae of concave-side inferior articular processes examined by Enneking and Harrington did not show evidence of hypertrophy due to increased loading. This suggests that the facet joints are not asymmetrically loaded in scoliosis, but does not rule out asymmetric vertebral body loading.

### Comment no. 10

I find Dr Stokes research very interesting because it deals with the biomechanical pathogenesis of idiopathic scoliosis that has been the basis of my treatment for scoliosis since 1995 [99-102]. Multilevel arthrodesis is not physiological and rehabilitation exercises made the scoliosis worse. Recently I have explained the biomechanical etiology, new rehabilitation treatment and prophylaxis of the so-called idiopathic scoliosis [101,102].

#### Response

Mechanical asymmetries superimposed on the strictly secondary effects analysed in the vicious cycle model would augment the calculated rate of progression that it predicts. Primary abnormalities, such as those proposed in the commentary could be included in the model, providing the magnitude of their effects were known as a quantitative estimate of the associated altered spinal loading.

## Plane of deformity – 2D method and need for a 3D model

### Comment no. 11

Dr Stokes' study relates to vertebral translation solely in the *frontal plane*. There is evidence that curve initiation in scoliosisis 3-D, and that it starts and develops simultaneously in the three cardinal planes [103].

### Comment no. 12

Even though the simulation is 2D the underlying biomechanical modulation process could act similarly in 3D. I exhortDr Stokes to expand his model to 3D.

#### Responses

Only the frontal plane was modeled. The natural history of curvatures in other planes is less well-known. Also, the magnitude of the frontal plane asymmetry is the largest in scoliosis. The risk of expanding the model to 3-D is that the assumptions would probably overwhelm the available data. Subject to this limitation, it would still be possible to use this approach to examine estimates of the line of action of the intervertebral forces in the sagittal plane. If these resultant forces were found to be displaced from the vertebral centers (*i.e*. not *'Follower loads' *[104]), then the *vicious cycle hypothesis *would predict growth modulation and consequential changes in the sagittal plane curves also.

## Simulation of lumbar scoliosis

### Comment no. 13 – *primary and secondary curves*

Is the lumbar spine deformity studied by Dr Stokes considered to be a *primary *scoliosis curve? If so what is the definition of primary and secondary curves fromanatomo-pathological and biomechanical – not clinical – points of view?

#### Response

The modeled geometry was averaged from stereoradiographic studies of 15 patients with a thoracolumbar Cobb angle in the range 27–43 degrees. The analysis did not take into consideration whether the curve was 'primary' or 'secondary', and only vertebral asymmetric growth (not disc wedging) was addressed. In this kind of analytical study, primary and secondary curves might differ, dependent on the relative amounts of vertebral and disc wedging in the curve

### Comment no. 14 – *applicable to thoracic curves?*

The results are based on computations relating to the anatomy of the lumbar spine that is free from support other than by elastic structures. Are the conclusions valid for the thoracic spine? The thoracic spine is supported laterally by ribcage and anteriorly by sternum and pressure of intra-thoracic organs [Moderator see Refs. [52,105-108]].

#### Response

The lumbar spine was modeled here since I consider that it is biomechanically less complex (it has fewer unknowns), so the model should be more valid. The conclusions apply quantitatively to the lumbar spine, but presumably also apply qualitatively to the thoracic spine, since the underlying spinal biomechanics and vertebral growth response to sustained altered loading are probably similar throughout the spine. Modeling (or otherwise determining) the forces acting on the thoracic spine provides a challenge for the future [Moderator see Comment no. 43 relating to a molecular classification for AIS].

## Buckling, axial loading, gravity and spinal growth force

### Comment no. 15

Millner and Dickson [17] state that for a progressive deformity to occur a buckling process resulting from biological factors during spinal growth is needed [Moderator see Ref. [109]]. Does Dr Stokes subscribe to the concept of such *'spinal buckling'*?

#### Response

*Buckling *is a term that normally refers to an instability producing catastrophic and sudden collapse. Such a collapse may be reversible if all deformations occur in the elastic range of tissue behavior. These events do not occur in scoliosis. I consider that the mechanism of mechanical modulation of growth is a more plausible explanation of scoliosis progression over time, prior to skeletal maturity.

### Comment no. 16

Is axial compressive loading the only force causing the 3-D translation of the vertebral-disc units of the apical segment and therefore determining the progression or not of the curve?

#### Response

Compressive forces are known to modulate growth in growth plates, but there might be other intervertebral force components such as shear forces acting on the discs and growth plates that act as 'driving forces' in the curve development. However, the available evidence indicates a lesser (and possibly negligible) bone growth modulation in response to altered shear [110]. The compressive forces acting on vertebrae and discs result from gravity forces as well as the forces from muscles that cross the spine. The forces due to muscle activation are generally of greater magnitude than forces due to the superimposed bodyweight [Moderator see Ref. [52]]. However, the unbalanced forces that result from trunk asymmetry might require additional muscle activation to maintain equilibrium, and hence add to the asymmetrical loading of the spine. The complexities associated with unbalanced trunk mass distribution and consequential asymmetrical loading are not specifically included in the present analysis. Each possible component of external loading (forces and moments in each of six principal directions) was analysed, and its possible contribution to curve progression was considered individually, as well as being averaged with the assumption that an individual exerts on average equal amounts of effort in each of the principal directions. Better understanding of body habitus, habitual activity and posture could be used to refine this assumption.

### Comment no. 17

Kawabata et al [111] in a mathematical study tested the hypothesis that when some vertebral bodies and discs outgrow their surrounding soft tissues, such as the ligaments and dura, a non-physiological *growth force *is created acting to restrain, buckling most easily when the *growth force *was localized from T9-L1. Suggested tethering of spinal growth by posterior musculoskeletal structures is an example of the action of a putative *growth force *altering spinal geometry [112]. Has Dr Stokes considered mechanical forces created by the linear growth of endplate physes?

#### Response

The suggestion that forces generated by growth and tethering contribute to the curve progression is very interesting, and this effect would augment the curve progression rate estimated here. However, in the present state of knowledge about soft tissue growth and remodeling, quantitative modeling of such effects would be difficult and entirely speculative [Moderator see Ref. [113]].

## Muscle activation strategies

### Comment no. 18

Dr Stokes suggests that "...different individuals may adopt differing muscle activation strategies", with consequences for the spinal loading that could explain why some individuals have more progressive scoliosis curves than others." Is there any evidence for the concept that muscle activation strategies can create minimal spinal loading asymmetry in smaller lumbar idiopathic curves? Is it not more likely that the *'muscle activation strategies' *are the expression of undetected neuromuscular dysfunction as the primary pathomechanism of AIS?

#### Response

The idea that differing *neuromuscular activation strategies *can be adopted, and lead to different degrees of spinal loading asymmetry and hence curve progression is a speculation. It was explored in the muscle force analyses [88]. These analyses predicted that forces generated by (or applied to) the trunk during normal function can result in asymmetrical spinal loading that tends to reverse the scoliosis, depending on how the muscles are activated. However, the curve-correcting neuromuscular activation strategy requires greater energy expenditure than that which produces curve worsening. Certainly one can speculate that there are pre-existing (morbid) muscle asymmetries, (though I am not aware of any measurements demonstrating it). My analyses deal only with the muscle forces required to satisfy the laws of physics (for static equilibrium).

### Comment no. 19

Are we really to believe that healthy adolescents spontaneously generate asymmetrical growth and deformity by inappropriate muscle strategies?

#### Response

My proposition is that once a scoliosis reaches a certain magnitude, then the laws of physics (asymmetric forces) and of physiology (mechanical modulation of growth) take over. Then, curve progression would occur in a person who is healthy and whose spine is responding in a normal physiological manner.

### Comment no. 20

I cannot agree that scoliosis is a result of spinal loading asymmetry depending on muscle activation strategies. In my view the asymmetry in development and growth of the spine and later scoliosis is caused by *movement asymmetry of both hips during gait *and because of the habit of standing on the right leg [99-102]. Primarily there are no "different muscle activation strategies" but these may occur later. Of course the curve apex is the most active in development of scoliosis because the largest deforming forces react here.

#### Response

A key underlying assumption is that loading sustained over a substantial proportion of the day modulates endochondral bone growth, whereas transient loading does not. As a consequence of this assumption, I expect that motion patterns in selected activities such as standing and walking probably do not generate the sustained abnormal loading required to alter a deformity as a result of altered skeletal growth.

## Treatment

### Comment no. 21

Dr. Stokes illustrates the importance of neuromuscular action in the bone growth modulation process so underlying both the roles of muscle strength and CNS motor control strategies as often claimed in conservative treatment.

### Comment no. 22

The hypothesis of the simulation explains findings we are recording and documenting at our rehabilitation center when unbalanced posture is corrected in scoliotic patients. Scoliosis curve progression ceases or regresses after applying 1) a raise to compensate for structural leg length discrepancy [Moderator see Ref. [114]], and 2) conservative treatment to stimulate a symmetrical spine and trunk muscles activation with control in both frontal and sagittal planes [115].

#### Response

Leg length inequality and its correction by shoe raise appears logical, but I understand that many scolioses associated with structural leg length inequality are 'postural' and inherently non-progressive [Moderator see Refs. [116-118]].

### Comment no. 23

The conclusion that we should now resurrect exercise programs and brace treatment for adolescent idiopathic scoliosis is a step too far [Moderator see Refs. [32-38,119]]. They only appear to work when applied to groups of predominantly post-pubertal adolescents with non-progressive scoliosis. If unloading the spine does not benefit young, surgically-treated early-onset scoliosis, where the spine is certainly "unloaded," why should we suppose it will do so later and by less robust means?

#### Response

As discussed in the response to Comment no. 24 the idea that exercise programs might be used to alter the course of growth and scoliosis progression appears theoretically plausible. But equally, the degree of 'plasticity' of the neuromuscular control system is probably insufficient to permit any useful amount of benefit.

### Comment no. 24

What suitable muscle rehabilitation programs might alter the prevailing spinal loading?

#### Response

Hypothetically, rehabilitation programs that would alter the muscle forces and hence the spinal loading over a substantial proportion of the time could alter the growth modulation effect. I speculate that this would require asymmetric muscle hypertrophy and strengthening, and/or development of new neuromuscular activation patterns. Alternatively, a brace that holds the spine in a less curved posture might result in less asymmetrically loaded spine. It seems possible that better understanding of these principles could improve therapeutic approaches that have been ineffective in the past.

### Comment no. 25

Although the author has summarized several clinical implications of his data, how can the findings be used to direct clinical approaches to reverse spinal curvatures in the early stages and/or prevent progression?

#### Response

I would certainly encourage clinicians to design therapeutic approaches to exploit these biomechanical explanations of scoliosis progression in treatment of small curves. One might aim either to modify the vertebral growth asymmetrically, or to modify the forces acting on the spinal column and endplate physes – both subject to the presence of sufficient residual growth. In order to comply with the requirement to 'do no harm', one ought to have the ability to predict which curves are likely to be progressive (to avoid treating benign curves). Prognosis of progressive curves is the greatest challenge to clinical application.

### Comment no. 26

The vicious cycle model applies equally to all etiologies of scoliosis. Dr Stokes has done the bridge work with animals and cross-checked in humans and developed systems that can be taken into the laboratory to do the molecular biology on what's happening at the cellular and tissue level. His model offers a context for generating *molecular *and *genomic *predictions, and thereby for bridging clinical and basic science in a meaningful way. Efforts to define how mechanical loading causes curve progression are now warranted. The molecular mechanism could be different in different subjects with scoliosis. Treatment may then be possible to block the vertebral cellular response to load [Moderator see Comment no. 43 Moderator's comment].

#### Response

Certainly, my studies analyse consequences of the observed phenomena, and do not explore the underlying physiology of growth modulation, so much work remains prior to a rationalized clinical application. Of course, this does not preclude empirical approaches subject to '*primus, non nocere*'.

## Leg length inequality and pelvic tilt scoliosis

### Comment no. 27

Structural leg length inequality of 2 cm or more is frequently observed as pelvic tilt scoliosis in adolescents attending scoliosis clinics [116-118,120,121]. The associated lumbar curves are compensatory and non-progressive with minimal structural features [122]. The inevitable asymmetric loading on the spine does not usually lead to curve progression. Why not? What are the loads so created? Could it be that in progressive lumbar AIS a genetic vulnerability of lumbar endplate physes to eccentric loading is a pre-requisite for curve progression? [Moderator see Comments no. 41 & 42].

#### Response

I speculate on three reasons why scoliosis curves associated with *anisomelia *are generally non-progressive: (1) A small proportion of the day is spent standing, hence the biomechanical effect is temporary; (2) The associated scoliosis curve is less than the threshold where the mechanically modulated growth would cause progression; and (3) If structural leg length inequality is acquired close to, or after skeletal maturity with little or no residual growth, then the growth modulation effect would not apply.

### Comment no. 28

Taking into account Dr Stokes' studies of modulation of both vertebrae and tibial growth it seems likely that lower limb bones are also affected by loading-compression modulation.

#### Response

Yes, I hope that the findings on tibial growth plates will be applicable to improved understanding and treatment of 'idiopathic' growth-related deformities of the proximal tibia (notably Blount's tibia vara).

## Vertebral body slenderness and non-standard vertebral rotation

### Comment no. 29 – *vertebral body shape*

A gender difference for normal *vertebral body shape *(height-width indices) [123,124] and spine slenderness [125] supports a role for spinal slenderness in progressive AIS [17,22,125,126] where females predominate and less so in curve initiation where the female-to-male prevalence is similar. When curves are greater than 30 degrees progression may be similar in boys and girls [127]. Has spinal slenderness been evaluated in Dr Stokes' model? How does Dr Stokes explain the female susceptibility to curve progression in AIS?

#### Response

This model (subject to its basic assumptions) would be able to predict the effects of differences in spinal slenderness as well as other gender dimorphisms (*e.g*. differing growth velocity profiles) as factors possibly explaining female predominance of progressive idiopathic scoliosis. Thank you for the suggestions for future simulations!

### Comment no. 30 – *non-standard vertebral rotation*

How does Dr Stokes account for *non-standard vertebral rotation *in lumbar and thoracic adolescent idiopathic scoliosis? [128,129].

#### Response

Being a two-dimensional analysis of vertebral wedging, my model is not able to explain either standard or non-standard vertebral rotation. Certainly, if biomechanical factors do explain the evolution of a scoliosis in the frontal plane, then biomechanics might also explain the associated rotational asymmetry that normally correlates with the frontal plane curvature [Moderator see Ref. [113]]. Unfortunately, we know very little about the details of how the loads are transmitted through the spine in three-dimensions.

## Sagittal curves and vertebral loading

### Comment no. 31

What is the role of mechanical forces in determining the *sagittal *curvatures of the normal spine? [Moderator see Ref. [52]]. In the normal sagittal alignment, slight anterior thoracic vertebral wedging is accepted as 'normal'. Surely there must be asymmetric forces acting across the sagittal plane of the endplate physes throughout the normal thoracic and lumbar spine in everyday life? In the normal thoracic kyphosis the center of gravity is anterior to the vertebral axis at the midthoracic location where the most common motion is into flexion. Carrying any weight adds asymmetric loads across the sagittal plane of the spine. Since the normal kyphosis is usually stable, how are these facts explained by the mechanical load theory in causing frontal plane spinal deformities?

#### Response

The *'Follower Load' *[104] is a concept that describes forces transmitted centrally through each articulation of the spinal column. This is the normal loading state, corresponding to absence of bending moments. For every spinal posture (curvature) the muscles should be activated to achieve the *'Follower Load' *condition. According to the growth modulation theory, any sustained failure to achieve this condition would lead to altered growth and vertebral wedging. The theory applies equally to the frontal and sagittal planes, but here is only explored for the frontal plane.

### Comment no. 32

With stereoradiographs it might be possible to determine the *plane of maximum deformity *by curve magnitude and location of the smallest height of concave apical vertebrae. If the *point of maximum loading *does not occur in the true frontal plane on the right or left side there may be a protective effect from the normal kyphosis with the curve looking less severe on the anteroposterior radiograph.

#### Response

Yes, the analysis could be made in any plane, including the plane of maximum deformity. Unfortunately, we lack information about the natural history of curve progression as seen in the plane of maximum deformity, most of the available information concerns the frontal plane [Moderator see Comment no. 31].

## Hypokyphosis and lateral vertebral loading

### Comment no. 33

In my clinical experience the most aggressive curves in severity and rate of change are those with *hypokyphosis*. While I understand the growth theories of Dickson, Roaf and Somerville [11,17,130] might not such patients have a more truly *lateral, or slightly postero-lateral, asymmetrical load *on the endplate physes that gives a more dramatic and true plane of maximum curvature on the anteroposterior radiograph?

#### Response

It is clear from this and other comments that my analyses of growth modulation and curve progression ought to be repeated in three-dimensions. This might give important insights into the variability of curve progression between individuals having differing spinal shape (*e.g*. hypokyphosis as a risk factor for progression). But I caution that the available data, especially those concerning neuromuscular activation might not yet support such analyses.

## Do neurogenic thoracic scolioses result from different skeletal pathomechanisms?

### Comment no. 34

Davids et al [131] write: "Thoracic apical segment lordosis ...has been identified as the central feature of the pathophysiology and pathomechanics of the progressive deformity associated with adolescent idiopathic scoliosis." [17]. Yet in presumed AIS they found that the most valuable single MRI indicator for abnormal central nervous system findings was the *absence *of a thoracic apical segment lordosis [131].

Questions:

a) May neurogenic thoracic scolioses initiate different skeletal pathomechanisms from those that evoke thoracic AIS?

b) Does the *vicious cycle hypothesis of pathogenesis *apply whether the curve is idiopathic or neurogenic?

#### Responses

The *'vicious cycle' *pathomechanism as modeled here -

a) Predicts a spinal morphology that depends on both the initial spinal geometry, and the prevailing pattern of spinal loading. Therefore, one would expect differing phenotypes to emerge, dependent on the exact form of both of these variables.

b) Assumes that pre-existing abnormal spinal curves (both scoliosis and kyphosis) will progress during adolescent growth whenever there is sustained altered spinal loading.

Yes, the concept is applicable whenever these two factors are present, as they are assumed to be present in both neurogenic and idiopathic cases.

## Curve progression without evident asymmetric loading

### Comment no. 35

In some conditions curve progression occurs without evidence to suggest that the cause is asymmetric loading:

a) In the child with *Prader-Willi syndrome *under good weight control with a mild scoliosis who, because of predicted short stature, receives growth hormone and the growth velocity suddenly increases at an age not normally expected and the curve rapidly increases. Is previous vertebral growth-plate damage being unmasked by the increased growth?

b) In *cardiac transplant patients *with a moderate scoliosis but poor nutritional status pre-transplant who characteristically have a sudden increase in their scoliosis post-transplant when they are nutritionally improved and physically more active.

I don't know if asymmetric forces within vertebrae are involved in these two examples. Basic questions are: How much might be mechanical forces? How much might be genetically pre-programmed growth-plate cartilage? Or, an interaction between the two? So many questions! Such a *'vicious cycle'*!

#### Response

A healthy person growing at a normal rate usually succeeds in creating an adequately symmetrical spine. We do not know all the regulatory mechanisms, but there would seem to be many ways that this process might go wrong.

## Relative anterior spinal overgrowth (*RASO*) phenomenon

### Comment no. 36

Dr Stokes does not mention the *RASO *phenomenon for progressive AIS. Anatomical studies have established that in structural scoliosis the anterior vertebral components are longer than the posterior elements [10,17,21,24,55,56,59]. This spinal growth disproportion is interpreted as resulting from r*elative anterior spinal overgrowth *(*RASO*) arising from endplate physeal activity from unknown causes, primary or secondary. According to the *RASO *concept the *initiation *and *some of the progression *of structural scoliosis is growth-induced with an unquantified role for mechanical factors with vertebral modeling occurring in accordance with the effects of Hueter-Volkmann and Wolff [2,132-134]. Goldberg et al [31] question the mechanical modulation concept of scoliosis curve progression.

Questions

a) Does Dr Stokes distinguish *initiating and progressive *factors in the causation of adolescent idiopathic scoliosis?

b) What creates the relative anterior spinal lengthening of structural scoliosis in the *'vicious cycle' *hypothesis?

c) In idiopathic scoliosis how much progression is biologic (*i.e. RASO*) and how much is mechanical?

d) May the proportion of biologic to mechanical factors contributing to curve progression differ not only by curve severity but also by curve type?

#### Responses

In the context of the *Relative Anterior Spinal Overgrowth (RASO) phenomenon*:

(a) Yes, the present paper concerns progression secondary to loading that is altered by a pre-existing curve. There must be a separate initiating factor.

(b) My own (albeit cross-sectional) studies of spinal shape in three-dimensions suggest that the curvatures in the frontal and sagittal planes do not develop synchronously (their magnitudes do not correlate)[90]. This argues that *RASO *might be a predisposing factor, not explainable by the growth modulation phenomenon. However, we need better natural history data to answer this question.

(c) This paper concludes that the vertebral growth modulation mechanism can explain curve progression of about 2 degrees (Cobb) per year over six years. This suggests that there are other mechanisms (possibly non-mechanical) that contribute to curve progression in cases of more rapid curve progression [Moderator see Comments no. 41 & 42].

(d) Different curve types may have differing tendency to progression, perhaps because of differing geometric factors, or because of differing relative contributions of mechanical and non-mechanical factors.

### Comment no. 37

The response to Comment no. 36 raises questions relating to the pathomechanisms that initiate a) the focal 3-D vertebral translation of AIS [103] – more often in adolescent females, and b) the kyphoses of Scheuermann' disease [135] – more common in males with its relative posterior spinal overgrowth (RPSO) combined with scoliosis in a few cases. I see an initiating ribcage asymmetry mechanism at work in a) and in the scolioses of b)[1].

#### Response

In the answer to Comment no. 36 it is proposed that initiating factors may be distinguishable from factors associated with progression. Initiating factors would remain constant, while factors that 'drive' the progression would correlate with magnitude of scoliosis. This leads me to view both hypokyphosis [17,90] and ribcage asymmetry [1,136] as possible *initiating factors*, since both appear to be present in scoliosis. But these abnormalities do not correlate with the magnitude of the scoliosis (in cross-sectional studies) and apparently do not increase during the adolescent progression phase.

## Some other biologic concepts of curve progression

### Comment no. 38 – *vertebral resorption by osteoclasts?*

Is asymmetrical growth at the scoliotic curve apex the only explanation for the concave vertebral wedging? In general the more severe the curvature the more the wedging. But in some very severe scolioses the concave height is shorter than expected given the age of the subject. This raises the question: is some of the wedging due to *bone resorption by osteoclasts? *Is there data to test whether loads not only reduce cartilaginous growth but also generate osseous remodeling? That may be Wolfish thinking but there is some data emerging in patients with solid congenital concave bars being distracted with Vertical Titanium Prosthetic Ribs [137] of an increase in length of the bar suggesting remodeling with longitudinal growth [Moderator see Ref. [63]].

#### Response

Yes, the present analysis excludes vertebral wedging resulting from asymmetric collapse of vertebral bone and loss of vertebral height in the convex side, as probably occurs in the aging, osteoporotic spine. Shape changes in the intervertebral disc (*e.g*. selective, concave side degeneration) might also contribute to curve progression after skeletal maturity.

### Comment no. 39 – *chronic cumulative effect of repetitive stresses?*

Stehbens [138] attributes the mechanical mechanism of curve progression to the *chronic cumulative effect of repetitive stresses *applied asymmetrically to the spinal postural deformity. This hypothesis implies that the biological mechanism may involve s*tress-activated protein kinases (SAPKs) *released in endplate physes. SAPKs are important regulators of a variety of repetitive loadings including tendons. They are evaluated by measuring c-Jun N-terminal kinase (JNK) activation [139] a signaling event in oxidative-stress-mediated cell death protected, or modulated by the selenium-containing antioxidant enzyme glutathione peroxidase [140]. Such stress-activation appears to be mediated through a calcium-dependent mechanotransduction pathway needing growth factors for mitogenesis [79]. Will Dr Stokes please comment on this repetitive disorder hypothesis.

#### Response

We have quantified growth plate response to altered load at the gross organ level, with some additional studies of cell numbers and rates of chondrocytic proliferation and hypertrophy [86,141]. However, I believe that knowledge about the specific pathways and growth plate chondrocyte regulatory mechanisms as they relate to altered mechanical environment is at an early stage [Moderator see Ref. [20]].

### Comment no. 40 – *platelet/skeletal hypothesis*

Emanating from the findings of Lowe et al [142,143] a recent hypothesis suggests that platelets activated in deforming immature vertebrae release growth factors that abet hormonal and mechanical factors to stimulate relative anterior spinal overgrowth and promote curve progression [96,144]. Will Dr Stokes please comment on this hypothesis?

#### Response

The vicious cycle model evaluated here supposes a mechanism of progression, without any abnormality of growth plate physiology. There is every reason to look for additional mechanisms whereby the mechanical stress-growth relationship might be altered systemically, or by local cell-cell interactive and regulatory mechanisms.

## Vertebral body growth – genetic and mechanical factors

### Comment no. 41

Ganey and Ogden [85] conclude that vertebral body growth relies not only on genetic factors but also responds to epigenetic factors including muscle tone, upright posture and activity. Vertebral body height in the mid-sagittal plane may be primarily genetically determined and relatively unaffected by mechanical factors associated with weight-bearing in the erect posture. In contrast, latitudinal and peripheral vertebral growth are more dependent on weight-bearing in the upright posture [145]. Ganey and Ogden suggest that the differential growth response of the peripheral *versus *the central portions of endplate physes of the spinal symphyses [146] may be a factor in the eccentric growth pattern that leads to the development and progression of structural scoliosis [Moderator This concept is developed by others in Comment no. 42].

#### Response

Overall skeletal growth (as evidenced by stature) as well as body proportions appear to be insensitive to mechanical influences associated with activity levels (young athletes are not shorter or taller), therefore genetic and nutritional factors appear to predominate. However, growth plates do respond to a sustained alteration in mechanical load, (*e.g*. altered muscle 'tone' could produce this effect). Within a growth plate, I am not aware of anatomical or structural differences that would make one region more sensitive than any other. I would look for regionally altered load, not regional differences within the endplate physes as a cause of unbalanced growth. [Moderator see Refs. [71,80,147]; Ratcliffe [71] speculates that AIS results from retarded growth on the affected side due to failure of the extra-osseous arterial supply during childhood and adolescence to compensate for the normal reduction of anastomoses between the arteries supplying the radial sectors of juxta-physeal regions of immature vertebrae].

## Vertebral symphyseal growth, type IX collagen and dysplasia

### Comment no. 42

Critical attention is required of:

1. The lack of an ossified epiphysis in human vertebral bodies [95], and

2. The vulnerable time for AIS curve initiation and progression is the period of spinal growth between closure of the neurocentral synchondroses at 6–7 years [148] and appearance of the ossified "ring" apophyses (11–14 years [85])

We attempt here to interpret such developmental features of the normal spine in relation to both the mechanically-induced matrix modeling of progressive AIS reported on by Dr Stokes and new discoveries of collagen structure. Theoretical analysis of the evidence leads us to the view that type IX collagen in disc tissues may have significance for the development of progressive AIS. In laboratory evaluations some disc tissues may have included vertebral endplate physis. The detection of defects in such matrices would be incendiary to the mechanical loading hypothesis of idiopathic scoliosis.

**Figure 3:**
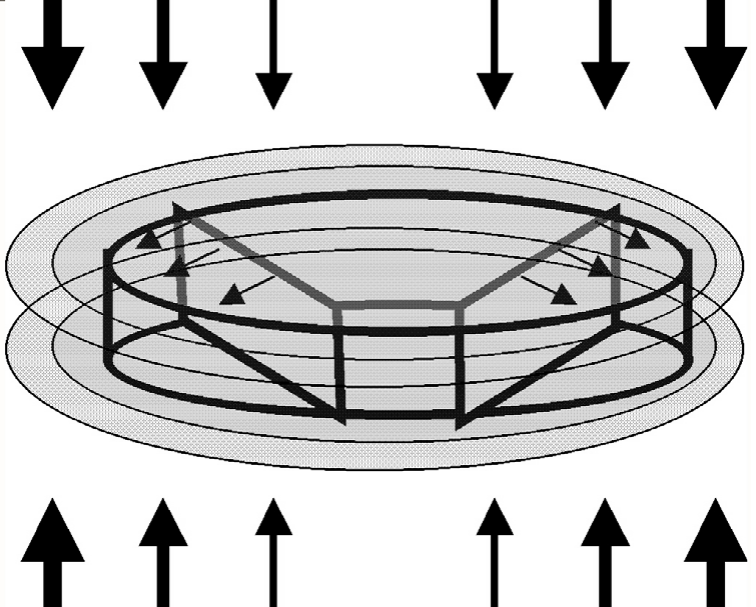
Diagram of vertebral body growth including that from the *neurocentral synchondroses (neurosomatic growth cartilages)*; the latter are shown by the smallest arrows as growing postero-laterally. The increase in dimensions of the vertebral body shown as a concentric (ellipsoid) simulates radial vertebral body growth. It is proposed that the increased girth of the vertebral body leads to increased axial loading indicated by the weights of the larger arrows placed above and below it.

*Putative dysplasia at the vertebral-disc interface under load*. We speculate that after the closure of neurocentral synchondrosis the vulnerability to AIS relates to: (1) peripheral annular insertions remodeling from genetic causes as a dysplastic phenotype; and (2) increasing loads with increasing vertebral size (Figure 3). In particular, a *dysplastic vertebral-disc interface *pre-empts normal turnover, accentuates shear dynamics at the *annular-vertebral body interface *and modifies discal properties.

The evidence for this novel concept includes:

1. The neurosynchondroses being bipolar contribute to both vertebral body and arch until the reserve zone cartilage is fully utilized (Figure 3). Each cartilaginous physis leaves sclerotic bone as a scar much the same as in a long bone [85], while the annular component of the disc shows remnants of a "biomechanical "wake interface between lamellae. The term "biomechanical wake" refers to the progressive transition of tissue and annular attachment that occurs in concert with the radial expansion of the vertebrae and disc. As the centrum and posterior arches have separate embryonic origins it is possible that the neurosynchondroses are of dual composition. To our knowledge there are no data to demonstrate regional mutational composition in a structure derived from two ontogenetic sources [Moderator see Ref. [149]].

2. Disc height does not change appreciably during growth (Figure 1). The increase of disc cross-sectional area is presumably derived from vertebral appositional growth with both central and peripheral areas of the disc subjected to increasing loads (Figure 3). The *central *annular insertions interface with the reserve zone cartilage of endplate physes while the *more lateral *annular insertions interface peripherally with the apophyseal "rings" – at first uncalcified, then calcified and later ossified [85] [Moderator see Ref. [95], Figure 1-A].

3. The mechanical strains from increasing loads with increasing vertebral body size will differ for *central *and *peripheral *annulus, dependent on anatomy that are likely to affect modeling and remodeling. In the rapidly growing adolescent years, *peripheral *annular tissues may be more susceptible to mechanical imbalance; that is the potential for anomaly increases during the expansion of the vertebral body particularly when loading is altered by deformity and posture.

4. The genetics of disc degeneration demonstrates the linkage of type IX collagen defects with its propensity to stabilize a collagen fibril network [150].

5. Type IX tryptophan Trp2 allele is associated with a predilection to disc degeneration including an increased prevalence of radial tears [151,152].

6. Knockout mice phenotypes for type IX collagen genes develop arthritic morphology that is sensitized to mechanical loading rather than overtly to matrix assembly [153-155]. An incidental anatomic characteristic of these mice is lack of ossified vertebral epiphyses [154].

7. Type IX collagen in humans differs in structure between the spine and articular cartilage, in particular by lacking the non-collagenous NC4 domain [156]. In articular cartilage this domain is critical and binds competitively to *cartilage oligomeric matrix protein (COMP)*, a protein that in aberrance affects matrix assembly [157-159].

8. Mutations in genes encoding for type IX collagen and COMP result in two related human bone dysplasias (pseudoachondroplasia and multiple epiphyseal dysplasia) each with early development of arthritis [160,161].

#### Response

In the analysis of a 'vicious cycle' presented here, it is assumed that: (1) at the outset, a pre-existing scoliosis curvature initiates the mechanically-modulated alteration of growth that causes worsening of the scoliosis; and (2) everything else is anatomically and physiologically 'normal'. Further, the analyses are based on available quantitative information concerning spinal loading and modulation of endochondral (longitudinal) growth of vertebrae. We lack quantitative data on how mechanical factors affect the modeling, remodeling and degeneration of the intervertebral disc and other spinal structures. Therefore, presumed mechanisms of discal deformity were not included in the present analyses. However, deformity of discs, as well as of vertebral shape are involved in the scoliosis deformity. I hope that future analyses will be able to include additional factors, including appositional bone growth, soft tissue changes, and any connective tissue abnormalities that can be identified and quantified.

## Mechanotransduction in articular cartilage, vertebral growth plates and other tissues and organs

### Comment no. 43

*Mechanotransduction *is the process by which cells convert mechanical energy into electrical or chemical signals [72,73,75]. It lies within the field of *mechanobiology *that in the skeleton includes the three effects of Hueter-Volkmann, Pauwels and Wolff [85,132-134]. (The *Pauwels' effect *is where intermittent pressure within the limits of physiological stress and strain stimulates the growth plates of a healthy bone [2,7]). In addition to studies on the intervertebral disc [162,163] there is much recent study of articular cartilage as efforts expand to discover disease-modifying drugs to treat or prevent osteoarthritis [164-167]. According to Ingber [73] mechanical signals may be integrated with other environmental signals – including growth factors and extracellular matrix – and transduced into a biochemical response through force-dependent changes in scaffold geometry or molecular mechanics. Stoltz [168] states that in chondrocytes many genes are regulated up and down by mechanical forces and the response depends not only on the duration and amplitude of the forces, but also on their variations in time. Lammi [169] reviewing articular cartilage states that possible mechanotransduction pathways in chondrocytes activated by load include the integrin-interleukin-4 route, NMDA receptors, and P2Y2 purinoceptors the latter involving ATP [170]. If such load-sensitive receptors are present in chondrocytes of endplate physes variation in gene expression by age, gender and topography may underlie the vulnerability to curve progression under eccentric load. There is preliminary work evaluating the effect of mechanical loads on mRNA expression of rat tibial growth plate cells [20]. Are any of the current methods now being used to study articular cartilage mechanotransduction being applied to endplates?

#### Response

The mechanical influences on articular chondrocytes and extra-cellular matrix synthesis and degradation are reviewed in Grodzinsky et al [171]. However, I suspect that little of this information can be applied to the very different growth plate chondrocytic phenotype characterized by high rates of proliferation (cycle time about 48 hours), rapid hypertrophy and abundant matrix synthesis, and eventual apoptosis. The mechanisms of *mechanotransduction *and their effects on cells in each stage of this differentiation cascade (and the rate of differentiation itself) are probably very specific to growth plate chondrocytes.

*Moderator: *In addition to skeletal tissues, muscles, tendons and ligaments [78,79], mechanotransduction is involved in the senses of touch, balance (spindle receptors and proprioceptors), hearing, baroreceptors (blood pressure), vascular remodeling from fluid shear stress [172] and systemic osmolarity [74]. Little is known about how mechanical input forces delivered to a cell result in a repertoire of output physiologic responses [74,75,172] though recently force-transducing molecules – *mechanosensitive ion channels *– have been identified in cell membranes with lipids [74] and calcium channels in osteoblasts [173] intimately involved. In certain connective tissues mechanotransduction appears to involve cyclical mechanical strain upregulating extracellular matrix genes suggesting that such genes are possible targets for novel therapeutic intervention [81].

*Melatonin-signaling defect, P factor and possible medical treatment for scoliosis*. In progressive AIS, Moreau and colleagues in 2004 reported that melatonin-signaling transduction is impaired in vertebral osteoblasts and other cells by the inactivation of Gi proteins [174]. Their 2006 scientific presentations showed this to be associated with high levels of a circulating protein P factor that appears essential for the initiation and progression of AIS through a specific signaling action during a postnatal window (175,176). In support of this interpretation 45% of melatonin-deficient mice rendered bipedal developed scoliosis but not after being genetically modified to be devoid of P factor or its receptor [175,176]. These findings reveal a molecular classification for AIS, suggest innovative diagnostic tools and the prospect of tailored pharmacological approaches to rescue the melatonin-signaling defect [177] (? aggravated by estrogens [178]). A systemic abnormality of cell differentiation is proposed as a novel mechanism in the etiopathogenesis of AIS [68] but how this may relate to vertebral growth and mechanical loads is not clear [Moderator see Comment no. 26]. The melatonin-signaling defect and the P factor excess could be the pathomechanism leading to the *pre-existing scoliosis curve *of Dr Stokes' vicious cycle hypothesis of pathogenesis. Another pathomechanism, albeit speculative, is vertebral symphyseal dysplasia [Moderator see Comment no. 42]. A possible role for anomalous left-right asymmetries in AIS etiopathogenesis [179] is suggested by evidence detected in the ribs [1], appendicular skeleton [22,120,121,180,181] and brain [182].

## Is the adjective 'vicious' appropriate?

### Comment no. 44

Curve progression is usually not *'vicious' *in that most small curves of 20 degrees or less revealed by school screening stabilize and are benign and may even resolve. In accordance with the concept of Asher and Burton [183] might not a better description be "*the growth-induced torsion (and counter-torsion) concept*" [22] implying that growth is the major factor but allowing a place for its modulation by mechanical forces through ill-understood biologic mechanisms? [20].

#### Response

The adjective 'vicious' appears entirely consistent with the definition of *'vicious circle' *('pair or series of evils that intensify each other by reaction')[184]. Here the pair of 'evils' would be (1) sustained, asymmetric loading of the spine and (2) lateral spinal curvature. They interact in an accelerating (intensifying) interdependency in the present hypothesis.

## Prognosis

### Comment no. 45

While variables that predict whether curves are progressive or non-progressive have been examined clinically in relation to prognosis with some success [26,27,65,127,185-189] the biologic mechanisms that determine progression, stabilization, or resolution of AIS curves are unknown and deserve more study [18,20] [Moderator see Refs. [68,174-178]].

#### Response

I agree that the urgent challenge is to be able to distinguish the factors that predict whether a curve is progressive or not. Analytical models can be very useful tools to examine quantitatively 'what if' scenarios, and to eliminate options that are found to be implausible.

## References

[B1] Sevastik JA, Burwell RG, Dangerfield PH (2003). A new concept for the etiopathogenesis of the thoracospinal deformity of idiopathic scoliosis: summary of an electronic focus group debate of the IBSE. Eur Spine J.

[B2] Mau H (1984). Specifizierung der korrespondierenden Wachstums-Gesetze von Hueter-Volkmann und Pauwels (Wachstumdeformitäten) und ihre Beziehung zu den Belasungsdeförmitäten. Z Orthop.

[B3] Stokes IAF, Spence H, Aronsson DD, Kilmer N (1996). Mechanical modulation of vertebral body growth: implications for scoliosis progression. Spine.

[B4] Stokes IAF, Burwell RG, Dangerfield PH, Lowe TG, Margulies JY (2000). Hueter-Volkmann effect. Etiology of Adolescent Idiopathic Scoliosis.

[B5] Arkin AM (1949). The mechanism of structural changes in scoliosis. J Bone Joint Surg [Am].

[B6] Arkin AM, Katz JF (1956). The effects of pressure on epiphyseal growth. The mechanism of plasticity of growing bones. J Bone Joint Surg [Am].

[B7] Pauwels F (1975). Eine klinische Beobachtung als Beispiel und Beweis für funtionelle Anspassung des Knochens durch Längenwachstum. Z Orthop.

[B8] Risser JC (1949). The mechanism of structural changes in scoliosis. Discussion after the paper of Arkin AM. J Bone Joint Surg [Am].

[B9] Roaf R (1958). Rotation movements of the spine with special reference to scoliosis. J Bone Joint Surg (Br).

[B10] Roaf R (1960). Vertebral growth and its mechanical control. J Bone Joint Surg (Br).

[B11] Roaf R (1966). The basic anatomy of scoliosis. J Bone Joint Surg [Br].

[B12] Burwell RG (1971). The relationship between scoliosis and growth. Scoliosis and Growth Proceedings of a Third Symposium: 13 November 1970.

[B13] Valentin B (1991). Geschicte der Orthopädie.

[B14] Kohler R, Kohler R, Picault C (1992). An historical survey of treatment of scoliosis. Proceedings of the European Spinal Deformities Society: 17–19 June Lyon, France.

[B15] Burwell RG, Cole AA, Cook TA, Grivas TB, Kiel AW, Moulton A, Thirlwall AS, Upadhyay SS, Webb JK, Wemyss-Holden SA, Whitwell DJ, Wojcik AS, Wythers DJ (1992). Pathogenesis of idiopathic scoliosis: the Nottingham concept. Acta Orthop Belg.

[B16] Perdriolle R, Becchetti S, Vidal J, Lopez P (1993). Mechanical process and growth cartilages. Essential factors in the progression of scoliosis. Spine.

[B17] Millner PA, Dickson RA (1996). Idiopathic scoliosis: biomechanics and biology. Eur Spine J.

[B18] Urban J, Stokes IAF (1999). Regulation of spinal growth and remodeling. Research into Spinal Deformities 2.

[B19] Veldhuizen AG, Wever DJ, Webb PJ (2000). The aetiology of idiopathic scoliosis: biomechanical and neuromuscular factors. Eur Spine J.

[B20] Villemure I, Chung MA, Seck CS, Kim MH, Matyas JR, Duncan NA, Grivas TB (2002). The effects of mechanical loading on mRNA expression of growth-plate cells. Research into Spinal Deformities 4 Studies in Health Technology & Informatics.

[B21] Villemure I, Aubin CE, Dansereau J (2002). Simulation of progressive deformities in adolescent idiopathic scoliosis using a biomechanical model integrating vertebral growth modulation. J Biomech Engin.

[B22] Burwell RG (2003). Aetiology of idiopathic scoliosis: current concepts. Pediatr Rehabil.

[B23] Castro FP (2003). Adolescent idiopathic scoliosis, bracing, and the Hueter-Volkmann principle. Spine J.

[B24] Villemure I, Aubin CE, Dansereau J, Labelle H (2004). Biomechanical simulations of the spine deformation process in adolescent idiopathic scoliosis from different pathogenesis hypotheses. Eur Spine J.

[B25] Parent S, Newton PO, Wenger DR (2005). Adolescent idiopathic scoliosis: etiology, anatomy, natural history, and bracing. American Academy of Orthopaedic Surgeons Instructional Course Lectures.

[B26] Nachemson AL, Peterson L-E (1995). Effectiveness of treatment with a brace in girls who have adolescent idiopathic scoliosis. A prospective, controlled study based on data from the Brace Study of the Scoliosis Research Society. J Bone Joint Surg [Am].

[B27] Peterson L-E, Nachemson AL (1995). Prediction of progression of the curve in girls who have adolescent idiopathic scoliosis of moderate severity. Logistic regression analysis based on data from the brace study of the Scoliosis Research Society. J Bone Joint Surg [Am].

[B28] Dickson RA, Weinstein SL (1999). Bracing (and screening) – yes or no?. J Bone Joint Surg [Br].

[B29] Wiley JW, Thomson JD, Mitchell TM, Smith BG, Banta JV (2000). Effectiveness of the Boston brace in treatment of large curves in adolescent idiopathic scoliosis. Spine.

[B30] Goldberg CJ, Moore DP, Fogarty EE, Dowling FE (2001). Adolescent idiopathic scoliosis: the effect of brace treatment on the incidence of surgery. Spine.

[B31] Goldberg CJ, Moore DP, Fogarty EE, Dowling FE, Tanguy A, Peuchot B (2002). Adolescent idiopathic scoliosis: Is the search for aetiology constrained by the orthosis?. Research into Spinal Deformities 3 Studies in Health Technology & Informatics.

[B32] Rigo M, (Program Chairman) (2004). International Conference on conservative management of spinal deformities Barcelona 23–24 January 2004. Pediatr Rehabil.

[B33] Negrini S, Antonini G, Carabalona R, Minozzi S (2004). Physical exercises as a treatment for adolescent idiopathic scoliosis. Pediatr Rehabil.

[B34] Weiss HR, Weiss GM (2004). Prevalence of surgery in patients with adolescent idiopathic scoliosis (AIS) following conservative treatment – A meta analysis [abstract]. Pediatr Rehabil.

[B35] Rigo M, Quera G, Puigdevall N, Corbella C, Gil MJ, Martinez S, Villagrasa M (2004). Biomechanics of specific exercises to correct scoliosis in 3D[abstract]. Pediatr Rehabil.

[B36] Maruyama T, Kitagawa T, Takeshita K, Mochizuki K, Nakamura K (2004). Effectiveness of conservative treatment for idiopathic scoliosis – a combination of brace treatment and physical treatment [abstract]. Pediatr Rehabil.

[B37] Negrini S, Aulisa L, Ferraro C, Fraschini P, Masiero S, Simonazzi P, Tedeschi C, Venturin A (2005). Italian guidelines on rehabilitation treatment of adolescents with scoliosis or other spinal deformities. Euro Medicophys.

[B38] Weiss H-R, Negrini S, Rigo M, Kotwicki T, Hawes MC, Grivas TB, Maruyama T, Landauer F (2006). Indications for conservative management of scoliosis (guidelines). Scoliosis.

[B39] Nachemson A, Danielsson AJ, Hasserius R, Ohlin A Curve progression at least ten years after maturity in patients with moderate idiopathic scoliosis – a prospective comparison of observation or brace treatment. Scoliosis Research Society 41st Annual Meeting & Course September 13–16, 2006 Monterey, California, USA.

[B40] Wright JG, Swiontkowski MF, Heckman JD (2003). Introducing levels of evidence to the *Journal*. J Bone Joint Surg [Am].

[B41] Limb D, Hay SM, Limb D, Hay SM (2007). Introduction, using evidence-based medicine in orthopaedic surgery. The Evidence for Orthopaedic Surgery.

[B42] Betz RR, Kim J, D'Andrea LP, Mulcahey J, Balsara RK, Clements DH (2003). An innovative technique of vertebral body stapling for the treatment of patients with adolescent idiopathic scoliosis: a feasibility, safety, and utility study. Spine.

[B43] Betz RR, D'Andrea LP, Mulcahey MJ, Chafetz MJ, Ross S (2005). Vertebral body stapling procedure for the treatment of scoliosis in the growing child. Clin Orthop.

[B44] Wall EJ, Bylski-Austrow DI, Kolata RJ, Crawford AH (2005). Endoscopic mechanical spinal hemiepiphysiodesis modifies spinal growth. Spine.

[B45] Cunningham ME, Frelinghuysen PH, Roh JS, Boachie-Adjei O, Green DW (2005). Fusionless scoliosis surgery. Curr Opin Pediatr.

[B46] Braun JT, Akyuz E, Ogilvie JW (2005). The use of animal models in fusionless scoliosis investigations. Spine.

[B47] Braun JT, Akyuz E, Udall H, Ogilvie JW, Brodke DS, Bachus KN (2006). Three-dimensional analysis of 2 fusionless scoliosis treatments: a flexible ligament tether versus a rigid-shape memory alloy staple. Spine.

[B48] Braun JT, Hoffman M, Akyuz E, Ogilvie JW, Brodke DS, Bachus KN (2006). Mechanical modulation of vertebral growth in the fusionless treatment of progressive scoliosis in an experimental model. Spine.

[B49] Little DG, Song KM, Katz D, Herring JA (2000). Relationship of peak height velocity to other maturity indicators in idiopathic scoliosis in girls. J Bone Joint Surg [Am].

[B50] Goldberg CJ, Dowling FE, Fogarty EE (1993). Adolescent idiopathic scoliosis: Is rising growth rate the triggering factor in progression?. Eur Spine J.

[B51] Sanders JO, Browne R, McConnell S, Margraf S, Cooney T, Finegold D Maturity assessment and curve progression in girls with idiopathic scoliosis. Scoliosis Research Society 41st Annual Meeting & Course September 13–16, 2006 Monterey, California, USA.

[B52] Harrison DE, Colloca CJ, Harrison DD, Janik TJ, Haas JW, Keller TS (2005). Anterior thoracic posture increases thoracolumbar disc loading. Eur Spine J.

[B53] Edgar M, Burwell RG, Dangerfield PH, Lowe TG, Margulies JY (2000). Neural mechanisms in the etiology of idiopathic scoliosis. Etiology of Adolescent Idiopathic Scoliosis.

[B54] Lowe TG, Edgar M, Margulies JY, Miller NH, Raso VJ, Reinker KA, Rivard C-H (2000). Current concepts review: etiology of idiopathic scoliosis: current trends in research. J Bone Joint Surg (Am).

[B55] Guo X, Chau W-W, Chan Y-L, Cheng J-Y-C (2003). Relative anterior spinal overgrowth in adolescent idiopathic scoliosis. Results of disproportionate endochondral-membranous bone growth. J Bone Joint Surg [Br].

[B56] Guo X, Chau W-W, Chan YL, Cheng J-C-Y, Burwell RG, Dangerfield PH (2005). Relative anterior spinal overgrowth in adolescent idiopathic scoliosis – result of disproportionate endochondral-membranous bone growth? Summary of an electronic focus group debate of the IBSE. Eur Spine J.

[B57] Roth M (1968). Idiopathic scoliosis caused by a short spinal cord. Acta Radiol Diagn (Stockh).

[B58] Porter RW (2001). The pathogenesis of idiopathic scoliosis: uncoupled neuro-osseous growth?. Eur Spine J.

[B59] Chu WCW, Lam WWM, Chan Y-l, Ng BKW, Lam T-p, Lee K-m, Guo X, Cheng JCY (2006). Relative shortening and functional tethering of spinal cord in adolescent idiopathic scoliosis? Study with multiplanar reformat magnetic resonance imaging and somatosensory evoked potentials. Spine.

[B60] Burwell RG (2001). Comment to "The pathogenesis of idiopathic scoliosis: uncoupled neuro-osseous growth?" by Porter RW. Eur Spine J.

[B61] Roberts S, Menage J, Eisenstein SM (1993). The cartilage end-plate and intervertebral disc in scoliosis: calcification and other sequelae. J Orthop Res.

[B62] Roberts S, Caterson B, Urban JPG, Burwell RG, Dangerfield PH, Lowe TG, Margulies JY (2000). Structure and composition of the cartilage end plate and intervertebral disc in scoliosis. Etiology of Adolescent Idiopathic Scoliosis.

[B63] Yoshihara H, Kawakami N, Matsuyama Y, Inoh H, Imagama S, Ishiguro N (2005). A histomorphometric study of scoliosis in pinealectomized chickens. Spine.

[B64] Cheng JCY, Burwell RG, Dangerfield PH, Lowe TG, Margulies JY (2000). Osteopenia. Etiology of Adolescent Idiopathic Scoliosis.

[B65] Hung VWY, Qin L, Cheung CSK, Lam TP, Ng BKW, Tse YK, Guo X, Lee KM, Cheng JCY (2005). Osteopenia: a new prognostic factor of curve progression in adolescent idiopathic scoliosis. J Bone Joint Surg (Am).

[B66] Cheung CSK, Lee WTK, Tse YK, Lee KM, Guo X, Qin L, Cheng JCY (2006). Generalised osteopenia in adolescent idiopathic scoliosis – association with abnormal pubertal growth, bone turnover, and calcium intake. Spine.

[B67] Cheng JYC, Hung VWY, Lee WTK, Yeung HY, Lam TP, Ng BKW, Guo X, Qin l, Uyttendaele D, Dangerfield PH (2006). Persistent osteopenia in adolescent idiopathic scoliosis – longitudinal monitoring of bone mineral density until skeletal maturity. Research into Spinal Deformities 5, Health Technology and Informatics.

[B68] Bredoux R, Corvazier E, Dally S, Chaabane C, Bobe R, Raies A, Moreau A, Enouf J (2006). Human platelet Ca^2+^-ATPases New markers of cell differentiation as illustrated in idiopathic scoliosis. Platelets.

[B69] Ratcliffe JF (1980). The arterial anatomy of the adult human lumbar vertebral body: a microangiogaphic study. J Anat.

[B70] Ratcliffe JF (1981). The arterial anatomy of the developing human dorsal and lumbar vertebral body. A microangiographic study. J Anat.

[B71] Ratcliffe JF (1982). An evaluation of the intra-osseous arterial anastomoses in the human vertebral body at different ages. A microarteriographic study. J Anat.

[B72] Hamill O (1997). Special topic: molecular mechanisms of mechanotransduction. Annu Rev Physiol.

[B73] Ingber DE (1997). Tensegrity: the architectural basis of cellular mechanotransduction. Annu Rev Physiol.

[B74] Kung C (2005). A possible unifying principle for mechanosensation. Nature.

[B75] Syntichaki P, Tavernarakis N (2004). Genetic models of mechanotransduction: The nematode *Caenorhabditis elegans*. Physiol Rev.

[B76] Frost HM (2003). Bone's mechanostat: a 2003 update. Anat Rec.

[B77] Lanyon LE, Stokes IAF (1981). Adaptive mechanics – the skeleton's response to mechanical stress. Mechanical factors and the skeleton.

[B78] Amiel D, Chu CR, Lee JL, Gordon SL, Blair SJ, Fine LJ (1995). Effect of loading on metabolism and repair of tendons and ligaments. Repetitive motion disorders of the upper extremity.

[B79] Banes AJ, Hu P, Xiao H, Sanderson MJ, Boitano S, Brigman B, Fischer T, Tsuzaki M, Brown TB, Almekinders LC, Lawrence WT, Gordon SL, Blair SJ, Fine LJ (1995). Tendon cells of the epitenon and internal tendon compartment communicate through gap junctions and respond differently to mechanical load and growth factors. Repetitive motion disorders of the upper extremity.

[B80] Würtz K, Neidlinger-Wilke C, Ignatius A, Wilke HJ, Claes L (2005). Cells from the distinct regions of the intervertebral disc differ in terms of their mechanosensitivity. Eur Spine J.

[B81] Kirwan RP, Fenerty CH, Crean J, Wordinger RJ, Clark AF, O'Brien CJ (2005). Influence of cyclical mechanical strain on extracellular matrix gene expression in human lamina cribrosa cells in vitro. Mol Vis.

[B82] Stokes IAF, Aronsson DD (2001). Disc and vertebral wedging in patients with progressive scoliosis. J Spinal Disord.

[B83] Stokes IAF, Windisch L (2006). Vertebral height growth predominates over intervertebral disc height growth in adolescents with scoliosis. Spine.

[B84] Dickson RA, Deacon P (1987). Annotation: Spinal growth. J Bone Joint Surg [Br].

[B85] Ganey TM, Ogden JA, Weinstein SL (2001). Development and maturation of the axial skeleton. The pediatric spine: principles and practice.

[B86] Stokes IA, Gwadera J, Dimock A, Farnum CE, Aronsson DD (2005). Modulation of vertebral and tibial growth by compression loading: diurnal versus full-time loading. J Orthop Res.

[B87] Urban MR, Fairbank JCT, Bibby SRS, Urban JPG (2001). Intervertebral disc composition in neuromuscular scoliosis: changes in cell density and glycosaminoglycan concentration at the curve apex. Spine.

[B88] Stokes IAF, Gardner-Morse M (2004). Muscle activation strategies and symmetry of spinal loading in the lumbar spine with scoliosis. Spine.

[B89] Stokes IAF, Aronsson DD, Cortright V, Beck S (2005). Endochondral growth in growth plates of three species at two anatomical locations modulated by mechanical compression and distraction. Orthop Res Soc.

[B90] Stokes IAF, Bigalow LC, Moreland MS (1987). Three-dimensional spinal curvature in idiopathic scoliosis. J Orthop Res.

[B91] Hughes RE, Chaffin DB, Lavender SA, Andersson GBJ (1994). Evaluation of muscle force prediction models of the lumbar trunk using surface electromyography. J Orthop Res.

[B92] D'Amico M, Roncoletta P, Grivas TB (2002). Joint segmental kinematic trunk motion and C.O.P. patterns for multifactorial posturographic analysis. Research into Spinal Deformities 4, Studies in Health Technology and Informatics.

[B93] Pfeiffer M, Pfeiffer D (2006). Important macroscopic and microscopic differences in the bony and cartilaginous regions adjacent to the lumbar intervertebral disc between animal and man: a caveat to overinterpretation of animal experiments. Eur Spine J.

[B94] Bylski-Austrow DI, Wall EJ, Rupert MP, Roy DR, Crawford AH (2001). Growth plate forces in the adolescent human knee: a radiographic and mechanical study of epiphyseal staples. J Pediatr Orthop.

[B95] Bick EM, Copel JW (1951). The ring apophysis of the human vertebra. Contribution to human osteogeny. II. J Bone Joint Surg (Am).

[B96] Burwell RG, Dangerfield PH (2006). Pathogenesis of progressive adolescent idiopathic scoliosis. platelet activation and vascular biology in immature vertebrae: an alternative molecular hypothesis. Acta Orthop Belg.

[B97] Mente PL, Aronsson DD, Stokes IAF, Iatridis JC (1999). Mechanical modulation of growth for the correction of vertebral wedge deformities. J Orthop Res.

[B98] Enneking WF, Harrington P (1969). Pathological changes in scoliosis. J Bone Joint Surg [Am].

[B99] Karski T (1998). Hip abductor contracture as a biomechanical factor in the development of the so-called "idiopathic scoliosis". Explanation of the etiology. Magyar Traumat Ortop.

[B100] Karski T, Grivas TB (2002). Etiology of the so-called "idiopathic scoliosis". Biomechanical explanation of spine deformity. Two groups of development of scoliosis. New rehabilitation treatment; possibility of prophylactics. Research into Spinal Deformities 4, Studies in Health Technology and Informatics.

[B101] Karski T (2005). Biomechanical explanation of etiology of the so-called idiopathic scoliosis. Two etiological groups – important for treatment and neo-prophylaxis. Pan Arab J Orth & Trauma.

[B102] Karski T, Uyttendaele D, Dangerfield PH (2006). Recent observations in the biomechanical etiology of so-called idiopathic scoliosis. New classification of spinal deformity – I-st, II-nd and III-rd etiopathological groups. Research into Spinal Deformities 5, Health Technology and Informatics.

[B103] Xiong B, Sevastik B, Sevastik J, Hedlund R, Dansereau J (1992). Early three dimensional radiographic changes in scoliosis. International Symposium on 3-D Scoliotic Deformities joined with the VIIth International Symposium on Spinal Deformity and Surface Topography.

[B104] Patwardhan AG, Havey RM, Meade KP, Lee B, Dunlap B (1999). A follower load increases the load-carrying capacity of the lumbar spine in compression. Spine.

[B105] Berg EE (1993). The sternal-rib complex. A possible fourth column of the spine. Spine.

[B106] Gardner ADH, Burwell RG, Dangerfield PH, Lowe TG, Margulies JY (2000). The significance of the sternum: the buttress of the thoracic spine. Etiology of Adolescent Idiopathic Scoliosis.

[B107] Watkins R, Watkins R, Williams L, Ahlbrand S, Garcia R, Karamanian A, Sharp L, Vo C, Hedman T (2005). Stability provided by the sternum and rib cage in the thoracic spine. Spine.

[B108] Burwell RG, Aujla RK, Cole AA, Dangerfield PH, Freeman BJC, Kirby AS, Pratt RK, Webb JK, Moulton A (2006). Supra-apical rib-vertebral and rib-spinal angle asymmetry are associated with curve parameters in preoperative adolescent idiopathic scoliosis (AIS): role of the sternal-rib complex (4^th ^column of spinal support)[abstract]. J Bone Joint Surg (Br) Orthop Proc.

[B109] Goto M, Kawakami N, Azegami H, Matsuyama Y, Tacheuchi K, Sasaoka R (2003). Buckling and bone remodeling as factors in the development of idiopathic scoliosis. Spine.

[B110] Moreland MS (1980). Morphological effects of torsion applied to growing bone. J Bone Joint Surg [Br].

[B111] Kawabata H, Ono K, Seguchi Y, Tanaka M (1998). Idiopathic scoliosis and growth – a biomechanical consideration. J Jap Orthop Assoc.

[B112] Wever DJ, Veldhuizen AG, Klein JP, Webb PJ, Nijenbanning G, Cool JC, v Horn JR (1999). A biomechanical analysis of the vertebral and rib deformities in structural scoliosis. Eur Spine J.

[B113] Kotwicki T, Napiontek M, Nawakowski A, Uyttendaele D, Dangerfield PH (2006). Transverse plane apical vertebra of structural thoracic curve: vertebra displacement versus vertebral deformation. Research into Spinal Deformities 5, Studies in Health Technology & Informatics.

[B114] Gurney B (2002). Review: Leg length discrepancy. Gait and Posture.

[B115] D'Amico M, Tanguy A, Peuchot B (2002). Scoliosis and leg asymmetries: a reliable approach to assess wedge solutions efficacy. Research into Spinal Deformities 3, Studies in Health Technology & Informatics.

[B116] Dickson RA (1983). Scoliosis in the community. Brit Med J.

[B117] Walker AP, Dickson RA School screening and pelvic tilt scoliosis. Lancet.

[B118] Manganiello A, Burwell RG, Dangerfield PH, Lowe TG, Margulies JY (2000). Lower limb length inequality and scoliosis. Etiology of Adolescent Idiopathic Scoliosis.

[B119] Stehbens WE (2003). Regression of juvenile idiopathic scoliosis. Exper Mol Path.

[B120] Burwell RG, Aujla RK, Freeman BJC, Dangerfield PH, Cole AA, Kirby AS, Pratt RK, Webb JK, Moulton A, Uyttendaele D, Dangerfield PH (2006). Patterns of extra-spinal left-right skeletal asymmetries in adolescent girls with lower spine scoliosis: relative lengthening of the ilium on the curve concavity and of right lower limb segments. Research into Spinal Deformities 5, Studies in Health Technology and Informatics.

[B121] Burwell RG, Aujla RK, Freeman BJC, Dangerfield PH, Cole AA, Kirby AS, Pratt RK, Webb JK, Moulton A, Uyttendaele D, Dangerfield PH (2006). Patterns of extra-spinal-spinal left-right skeletal asymmetries and proximo-distal disproportion in adolescent girls with lower spine scoliosis: ilio-femoral length asymmetry and bilateral tibial-foot length disproportion. Research into Spinal Deformities 5, Studies in Health Technology and Informatics.

[B122] Papaioannou T, Stokes I, Kenwright J (1982). Scoliosis associated with limb-length inequality. J Bone Joint Surg [Am].

[B123] Taylor JR, Twomey LT (1984). Sexual dimorphism in human vertebral body shape. J Anat.

[B124] Veldhuizen AG, Baas P, Webb PJ (1986). Observations on the growth of the adolescent spine. J Bone Joint Surg [Br].

[B125] Schultz AB, Sörensen S-E, Anderson GBJ (1984). Measurements of spine morphology in children, ages 10–16. Spine.

[B126] Skoglund LB, Miller JAA (1981). The length and proportions of the thoracolumbar spine in children with idiopathic scoliosis. Acta Orthop Scand.

[B127] Bunnell WP (1986). The natural history of idiopathic scoliosis before skeletal maturity. Spine.

[B128] Armstrong GWD, Livermore NB, Suzuki N, Armstrong JG (1982). Nonstandard vertebral rotation in scoliosis screening patients. Its prevalence and relation to the clinical deformity. Spine.

[B129] Burwell RG, Aujla RK, Dangerfield PH, Freeman BJC, Kirby AS, Webb JK, Moulton A (2006). Pelvic tilt scoliosis and lumbar idiopathic scoliosis in screening referrals: biplanar spinal pathomechanisms and frontal plane spinal tilt [abstract]. J Bone Joint Surg (Br) Orthop Proc.

[B130] Somerville EW (1952). Rotational lordosis: the development of the single curve. J Bone Joint Surg (Br).

[B131] Davids JR, Chamberlin E, Blackhurst DW (2004). Indications for magnetic resonance imaging in presumed adolescent idiopathic scoliosis. J Bone Joint Surg [Am].

[B132] Wolff J, Maquet P, Furlong R (1986). The Law of Bone Remodelling.

[B133] Wolff J (1988). The Classic. Concerning the interrelationship between form and function of the individual parts of the organism. Clin Orthop.

[B134] Golding JSR (1994). The mechanical factors which influence bone growth. Eur J Clin Nutr.

[B135] Deacon P, Berkin CR, Dickson RA (1985). Combined idiopathic kyphosis and scoliosis. An analysis of the lateral spinal curvatures associated with Scheuermann's disease. J Bone Joint Sirg (Br).

[B136] Stokes IAF, Dansereau J, Moreland MS (1989). Rib cage asymmetry in scoliosis. J Orthop Res.

[B137] Campbell RM, Smith MD, Mayes TC, Mangos JA, Willey-Courand DB, Kose N, Pinero RF, Alder ME, Duong HL, Surber JL (2004). The effect of opening wedge thoracostomy on thoracic insufficiency syndrome associated with fused ribs and congenital scoliosis. J Bone Joint Surg [Am].

[B138] Stehbens WE (2003). Pathogenesis of idiopathic scoliosis revisited. Exper Mol Path.

[B139] Arnoczky SP, Tian T, Lavagnino M, Gardner K, Schuler P, Morse P (2002). Activation of stress-related protein kinases (SAPK) in tendon cells following cyclic strain: the effects of strain frequency, strain magnitude, and cytosolic calcium. J Orthop Res.

[B140] Cheng WH, Zheng X, Quimby FR, Roneker CA, Lei XG (2003). Low levels of glutathione peroxidase 1 activity in selenium-deficient mouse liver affect c-JUN n-terminal kinase activation and p53 phosphorylation on Se-15 in pro-oxidant-induced aponecrosis. Biochem J.

[B141] Stokes IA, Mente PL, Iatridis JC, Farnum CE, Aronsson DD (2002). Enlargement of growth plate chondrocytes modulated by sustained mechanical loading. J Bone Joint Surg [Am].

[B142] Lowe TG, Lawellin D, Smith D, Price C, Haher T, Merola A, O'Brien M (2002). Platelet calmodulin levels in adolescent idiopathic scoliosis. Spine.

[B143] Lowe TG, Burwell RG, Dangerfield PH (2004). Platelet calmodulin levels in adolescent idiopathic scoliosis (AIS): can they predict curve progression and severity? Summary of an electronic focus group debate of the IBSE. Eur Spine J.

[B144] Burwell RG, Dangerfield PH (2003). Platelet calmodulin levels in adolescent idiopathic scoliosis. Do the levels correlate with curve progression and severity?[Letter]. Spine.

[B145] Taylor JR (1975). Growth of human intervertebral discs and vertebral bodies. J Anat.

[B146] Moore RJ (2000). The vertebral end-plate: what do we know?. Eur Spine J.

[B147] Heidari B, FitzPatrick D, Synnott K, McCormack D (2004). Modelling of annulus fibrosus imbalance as an aetiological factor in adolescent idiopathic scoliosis. Clin Biomech.

[B148] Lord MJ, Ogden JA, Ganey TM (1995). Postnatal development of the thoracic spine. Spine.

[B149] Burwell RG, Dangerfield PH, Sawatzky BJ (2004). Hypotheses on the pathogenesis of adolescent idiopathic scoliosis (AIS). X-chromosome mosaicism and microchimerism – need for appraisal in AIS?. International Research Society of Spinal Deformities Symposium.

[B150] Eyre DR, Pietka T, Weis MA, Wu JJ (2004). Co-valent cross-linking of the NC1 domain of collagen Type IX to collagen Type II in cartilage. J Biol Chem.

[B151] Kales SN, Linos A, Chatzis C, Sai Y, Halla M, Nasioulas G, Christiani DC (2004). The role of collagen IX in tryptophan polymorphisms in symptomatic intervertebral disc disease in Southern European patients. Spine.

[B152] Matsui Y, Mirza SK, Wu J-J, Carter B, Bellabarba C, Shaffrey CI, Chapman JR, Eyre DR (2004). The association of lumbar spondylolisthesis with collagen IX tryptophan alleles. J Bone Joint Surg [Br].

[B153] Aszódi A, Bateman JF, Gustafson E, Boot-Handford R, Fässler R (2000). Mammalian skeletogenesis and extracellular matrix: What can we learn from knockout mice?. Cell Structure & Function.

[B154] Fässler R, Schnegelsberg PNJ, Dausman J, Shinya T, Muragaki Y, McCarthy MT, Olsen BR, Jaenisch R (1994). Mice lacking á1(IX) collagen develop noninflammatory degenerative joint disease. Proc Natl Acad Sci USA.

[B155] Kimura T, Nakata K, Tsumaki N, Miyamoto S, Matsui Y, Ebara S, Ochi T (1996). Progressive degeneration of articular cartilage and intervertebral discs. An experimental study in transgenic mice bearing a type IX collagen mutation. Int Orthop.

[B156] Eyre DR, Matsui Y, Wu JJ (2002). Collagen polymorphisms of the intervertebral disc. Biochem Soc Trans.

[B157] Hecht JT, Hayes E, Haynes R, Cole WG (2005). COMP mutations, chondrocyte function and cartilage matrix. Matrix Biol.

[B158] Holden P, Meadows RS, Chapman KL, Grant ME, Kadler KE, Briggs MD (2001). Cartilage oligomeric matrix protein interacts with type IX collagen, and disruptions to these interactions identify a pathogenetic mechanism in a bone dysplasia family. J Biol Chem.

[B159] Pihlajamaa T, Lankinen H, Ylöstalo J, Valmu L, Jäälinoja J, Zaucke F, Spitznagel L, Gösling S, Puustinen A, Mörgelin M, Peränen J, Maurer P, Ala-Kokko L, Kilpeläinen I (2004). Characterization of recombinant amino-terminal NC4 domain of human collagen IX. Interaction with glysosaminoglycans and cartilage oligomeric matrix protein. J Biol Chem.

[B160] Lachman RS, Krakow D, Cohn DH, Rimoin DL (2005). MED, COMP, multilayered and NEIN: an overview of multiple epiphyseal dysplasia. Pediatr Radiol.

[B161] Unger SL, Briggs MD, Holden P, Zabel B, Ala-Kokko L, Paassilta P, Lohiniva J, Rimoin DL, Lachman RS, Cohn DH (2001). Multiple epiphyseal dysplasia: radiographic abnormalities with genotype. Pediatr Radiol.

[B162] Lotz JC, Hsieh AH, Walsh AL, Palmer EI, Chin JR (2002). Mechanobiology of the intervertebral disc. Biochem Soc Trans.

[B163] Urban JPG (2002). The role the physicochemical environment in determining disc cell behaviour. Biochem Soc Trans.

[B164] Eyre D (2002). Review: Collagen of articular cartilage. Arthritis Res.

[B165] Eyre DR, Wu JJ, Fernandes RJ, Pietka TA, Weis MA (2002). Recent developments in cartilage research; matrix biology of the collagen II/IX/XI heterofibril network. Biochem Soc Trans.

[B166] Stoltz J-F (2001). Mechanobiology: cartilage and chondrocyte. Volume 2; Biomedical and Health Research.

[B167] Stoltz J-F, Stoltz J-F (2004). Mechanobiology: cartilage and chondrocyte. Volume 3; Biomedical and Health Research.

[B168] Stoltz J-F, Stoltz J-F (2001). Concluding remarks and perspectives on cartilage and chondrocyte mechanobiology. Mechanobiology: cartilage and chondrocyte – Volume 2; Biomedical and Health Research.

[B169] Lammi MJ, Stoltz J-F (2004). Current perspectives on cartilage and chondrocyte mechanobiology. Mechanobiology: cartilage and chondrocyte – Volume 3; Biomedical and Health Research.

[B170] Millward-Sadler SJ, Wright MO, Flatman PW, Salter DM, Stoltz J-F (2004). ATP in the mechanotransduction pathway of normal human chondrocytes. Mechanobiology: cartilage and chondrocyte – Volume 3; Biomedical and Health Research.

[B171] Grodzinsky AJ, Levenston ME, Jin M, Frank EH (2000). Cartilage tissue remodeling in response to mechanical forces. Ann Rev Biomed Eng.

[B172] Tzima E, Irani-Tehrani M, Kiosses WB, Dejana E, Schultz DA, Engelhardt B, Cao G, DeLisser H, Schwartz MA (2005). A mechanosensory complex that mediates the endothelial cell response to fluid shear stress. Nature.

[B173] Haut Donahue TL, Genetos DC, Jacobs CR, Doanhue HJ, Yellowley CE (2004). Annexin V disruption impairs mechanically induced calcium signaling in osteoblastic cells. Bone.

[B174] Moreau A, Wang DS, Forget S, Azeddine B, Angeloni D, Fraschini F, Labelle H, Poitras B, Rivard C-H, Grimard G (2004). Melatonin signaling dysfunction in adolescent idiopathic scoliosis. Spine.

[B175] Moreau A, Boulanger H, Aubin C-E, Mathieu PA, Wang S, Bagnall K, Fairbank J Study of pathomechanisms initiating scoliotic deformities: identfication of a novel factor essential for the initiation and progression of scoliosis. Aetiology of Adolescent Idiopathic Scoliosis, 11th International Phillip Zorab Symposium, Christ Church, Oxford, UK, 3–5 April 2006 J Bone Joint Surg (Br) Orthop Proc.

[B176] Azeddine B, Boulanger H, Blain S, Limosani M, Aubin C-E, Mathieu PA, Labelle H, Poitras B, Rivard C-H, Grimard G, Ouellet JA, Bagnall KM, Moreau A Study of pathomechanisms initiating scoliotic deformities: identification of a novel factor essential for the initiation and progression of scoliosis. Scoliosis Research Society 41st Annual Meeting & Course September 13–16, 2006 Monterey, California, USA.

[B177] Moreau A, Azeddine B, Labelle H, Poitras B, Rivard C-H, Ouellet J, Grimard G, Fairbank J Functional and molecular classification of AIS: novel emerging concepts to understand its genetics causes and for the development of tailored pharmacological approaches. Aetiology of Adolescent Idiopathic Scoliosis, 11th International Phillip Zorab Symposium, Christ Church, Oxford, UK, 3–5 April 2006 J Bone Joint Surg (Br) Orthop Proc.

[B178] Letellier K, Azeddine B, Lacroix G, Wang SD, Turgeon I, Grimard G, Labelle H, Moreau A, Moldovan F Could estrogens be a key component of the pathogenesis of AIS?. Scoliosis Research Society 41st Annual Meeting & Course September 13–16, 2006 Monterey, California, USA.

[B179] Burwell RG, Freeman BJC, Dangerfield PH, Aujla RK, Cole AA, Kirby AS, Pratt RK, Webb JK, Moulton A Neurodevelopmental concept of maturational delay of the CNS body schema ("body-in-the-brain") for adolescent idiopathic scoliosis. British Scoliosis Society Annual Meeting 28–29th September 2006, Trinity College Dublin, Eire J Bone Joint Surg (Br) Orthop Proc.

[B180] Burwell RG, Aujla RK, Freeman BJC, Cole AA, Kirby AS, Pratt RK, Webb JK, Moulton A Iliac height asymmetry in adolescent girls with lower spine scoliosis: another finding of extra-spinal left-right skeletal length asymmetry. BRITSPINE Cardiff, UK, 26–28th April 2006 J Bone Joint Surg (Br) Orthop Proc.

[B181] Burwell RG, Aujla RK, Freeman BJC, Cole AA, Kirby AS, Pratt RK, Webb JK, Moulton A Thoracic scoliosis in screening referrals: apical vertebral rotation is associated with upper arm length asymmetry – interpretation and surgical implications. British Scoliosis Society Annual Meeting 28–29th September 2006, Trinity College Dublin, Eire J Bone Joint Surg (Br) Orthop Proc.

[B182] Liu T, Chu WCW, Li K, Yeung BHY, Guo L, Man GCW, Lam WWM, Wong STC, Cheng JCY Asymmetrical regional brain volume in adolescent idiopathic scoliosis girls – an MRI quantitative pilot study. British Scoliosis Society Annual Meeting 28–29th September 2006, Trinity College Dublin, Eire J Bone Joint Surg (Br) Orthop Proc.

[B183] Asher MA, Burton DC (1999). A concept of idiopathic scoliosis as imperfect torsion(s). Clin Orthop.

[B184] Fowler FG, Fowler HW (1969). Pocket Oxford Dictionary of Current English Usage.

[B185] Lonstein JE, Carlson JM (1984). The prediction of curve progression in untreated idiopathic scoliosis during growth. J Bone Joint Surg [Am].

[B186] Yamauchi Y, Yamaguchi T, Asaka Y (1988). Prediction of curve progression in idiopathic scoliosis based on initial roentgenograms. A proposal of an equation. Spine.

[B187] Weinstein SL (1999). Natural history. Spine.

[B188] Soucacos P, Zacharis K, Soultanis K, Gelalis J, Xenakis T, Beris AE (2000). Risk factors for idiopathic scoliosis: review of a 6-year prospective study. Orthopedics.

[B189] Weinstein SL, Weinstein SL (2001). Adolescent idiopathic scoliosis: natural history. The pediatric spine: principles and practice.

